# A Few Suggestions to Young Men Commencing or about to Commence the Practice of Dentistry

**Published:** 1853-10

**Authors:** J. F. Johnston

**Affiliations:** Indianapolis, Ind.


*A few Suggestions to Young Men Commenciag, or about to Commence the Practice of Dentistry.* By J. F. JOHNSTON, D. D. S., Indianapolis, Ind.

I do not expect in writing this article, to present to the profession, anything of a scientific character, or to develope new truths to the experienced; but on the contrary, state only that, which such men are familiar with, and which they can fully concur in; knowing thereby, it will have more weight with those for whom it is intended. I have not been in practice long enough, nor do I suppose the oldest practitioner has, to have forgotten the difficulties met and contended with in the start. The fact is, they are of such a nature, as to be indelibly fixed in the memory. I would not have the reader infer from this, that after he surmounts the first obstacles, his troubles cease; for if he should, he will be sadly misled. In the practice of our profession, the cases to be treated, being so various, (and this applies to surgical as well as mechanical dentistry), we find something new, and to a certain extent, perplexing in almost every operation ; which to perform successfully, requires zeal, untiring energy, and considerable versatility of talent. Yet, if from the beginning, these difficulties are met, with the proper determination to overcome them, those that occur subsequently, will annoy comparatively but little. The purport then, of this paper is, to prepare the beginner, and warn those about to begin, by calling their attention to certain professional trials, which are common in practice, but which may have escaped their attention. Pointing the former to the result, as to his future prospect, as also to the elevation or degredation of his profession, as depending upon his mode of practice ; thereby encouraging the worthy—for such will certainly aspire to do what others have done—and discourage, as we ought, those who are not ; and the latter, that they may not enter blindly into a profession, which, when they fully understand the nature of, they would discover perhaps, that they were totally unfit for. I was in a considerable extent, influenced to write this article, through being convinced from actual observation, not only of persons, but their operations, that many enter it, ignorant to an alarming extent, of what, in this age, is expected of them, or the amount of responsibility they assume. I hope this may, as it is intended, occasion such, to use vigorous efforts for improvement, or abandon it altogether. They ought to be made to feel, that honesty is a commendable trait of character, in this, as in other callings ; and also, that they have *animated* nature to operate upon. I have thought, that a thorough exposition, would, to a considerable extent, accomplish this, and would be the means of enlisting into the profession, only such men as were energetic, and have talent for it. The indolent will become alarmed, for they will learn, if they join us, they will have to work—and on this point I will dwell a little while ; for I feel sure, the thought of leading an easy life, has led many lazy fellows to style themselves dentists. To perform any operation well, the necessary amount of labor, time, and care must be bestowed. In the filling of a carious tooth, it will not do to wipe out the cavity with a little cotton, and stuff it as many do; but it must be thoroughly cleansed and properly shaped, and the stopping perfectly condensed and finished. The man who operates without taking these precautions, will have at the time quite a reputation, his patient not knowing what is necessary in such cases, will be led to remark how easy he operates; he don't cut, scrape, file, push, and polish all day, like Dr. A. or Dr. B., he is so gentle, and does it so quick. Let this distinguished individual go away, and come back in the course of a month—which by the way, he will be very careful not to do—and the note is changed. The talk will be something after this manner—well if Dr. A. or B. do hurt, file, and scrape, and polish for a long time, yet, there is a satisfaction in knowing, that it is not to be done over again, and that your teeth are saved. Now we have not only to submit to Dr. A. or B's mode of practice, but have it all to pay for again. Such is the effect of the indolent, and we might say dishonest man's practice; for he certainly can with all propriety, lay claim to both appellations. We cannot wonder then, at many doubting the efficacy of dental operations, or the suspicious look cast upon the stranger or beginner.

It will be seen, then, that neither carelessness or laziness will answer for a *respectable* dentist—so here allow me to say to those who expect to make fortunes without work, avoid the dental profession. Besides, it occurs to me, the practitioner ought to have some *little conscience*, and endeavor to give, as near as may be, value for that he receives. Ignorance, which we ought to be ashamed of, is the only excuse an honest man can make if he does otherwise. But none of us can be ignorant of neglect of duty. The beginner will find plugging perhaps the most difficult operation he will be called upon to perform, and will frequently be tempted to *overlook* a cavity that will be troublesome to fill. But I assure you, if you wish to succeed and elevate your profession and yourself with it, you will not do so. If your ambition did not prompt you to do what any one else can, would not your honor urge you to the task? Could you, for example, take a fee for filling a cavity in the crown of a molar tooth which your patient had pointed out to you, and leave unfilled a posterior approximal cavity in the same tooth, because it was difficult, and which, from its obscurity, your patient had at the present failed to discover? You certainly would not. And yet, although for the credit of the profession it is painful to record it, I know it is often done. Fortunately however, those who practice in that way, at last meet their reward. Tooth-ache will eventually ensue, and the patient will know who to thank for it. It is hardly necessary to add that the reputation of such is not enviable. But here, in justice to those engaged in this kind of practice, I must say I think many do so through timidity, arising from a lack of confidence in themselves more than any other cause, and avoid such operations in the fear of losing their reputation in the attempt to perform them. Such persons, however, are decidedly on the wrong side, and unfit for the profession. You must start out with the determination to do whatever is practicable. It is true it may annoy your patients at the time, be painful, and to them exceedingly tedious, and they would perhaps, as you are a beginner, imagine it roughly done, when in reality it was done in a manner equal to the best operators. But in the natural course of events, as is always the case when a man does his *duty* he will be properly appreciated, and afterwards his abilities will be no longer questioned. Such cases of neglect as the one just spoken of are common, and are met with by every one in practice. I frequently have such to treat, which, through carelessness in making examinations, or fear of being unsuccessful in the treatment, have been passed by. It seems to me I should feel very unpleasantly in receiving a fee for any operation performed, unless done to the best of my judgment, in the most perfect manner. Beginners through fear of offending, or losing business, should by no means deviate from what in their judgment, they conceive to be the proper course—doubtless they will be frequently urged to do so. They will often have patients who are egotistical enough to want all their own way. Such patients, if they won't submit to your plan of treatment, are better lost than retained. We will mention, as an example, a kind of circumstance that occurs to all. You are called upon to insert teeth in a mouth full of loose roots that have, by absorption, but little more than gum attachments; you advise, of course, as a preliminary step, their removal, and the mouth in every way to be restored to health, to which the party objects, asserting that Dr.——— offered to insert them a first rate set without having to submit to anything of the kind. They had learned you were a good dentist, and had determined to give you a trial, yet would have to return to Dr. ——— if you would not insert them over the roots. Now, it is true, it would be trying to let them go, especially if you were short of funds, and had but a very small practice. Nevertheless, you had far better do so. If you stay long enough in that place, (and it is best to drop down and stick, as a general thing,) it is quite probable this person will return to you—after having worn the teeth made by the Quack till they were no longer endurable—with regrets they did not employ you in the first place. Now would not your position be vastly better than if you had suffered yourself to be dictated to, and have followed the course of the emperic? You may rest assured the honest, conscientious course, is the one to be taken. You may be embarrassed at first, but you will finally succeed. Your patients will have the utmost confidence, will heed your advice, and when necessary to operate, will place themselves passively and fearlessly under your care.

I have been told of a certain dentist who occasionally preaches for the avowed purpose of saving the souls of his dear hearers; and after having discharged this christian duty, he very affectionately tells them he will save their teeth. It is supposed by some, he comes as near saving the former as the latter, and that he saves more of the people's *funds* than either. Such men degrade the profession perhaps more than any other class. They are so tender-hearted that they cannot refuse to practice according to the wishes of their patients, (unless it is decidedly out of pocket,) when they must know it is often entirely at variance with correct principles. Thus taking advantage of the credulity of the people, who imagine, because they appear to be pious men, they are too honest to practice dentistry, unless in the best manner. This means of obtaining business, is, however, by some condemned; I am one of that number. A dentist could, and should be a religious man; but I doubt if he should preach for a practice. To pursue, as I conceive, the proper course, you must to sum up all, commence with the expectation of waiting your time for a practice; knowing you must first have the *requisite qualifications*, then gain the confidence of the people, which you will surely do if you discharge your duties faithfully, and make it a point not to succumb to any thing that can be done, which properly comes under the dentist's care. And thus you will add lustre to your profession and gain for yourself an enviable reputation and a fortune honestly.

